# Supplementary material for: Patterns of Intron Gain and Loss in Fungi
Source: PLoS Biol. 2004 Nov 30;2(12):e422. doi: 10.1371/journal.pbio.0020422 (PMC532390; doi:10.1371/journal.pbio.0020422)
Supplement: Table S1 — Also available at http://genes.mit.edu/NielsenEtAl/. (4.3 MB ZIP). [file pbio.0020422.st001.zip › NielsenEtAl/html/97.html]

AN4684.1.NCU02148.1.MG02622.1.FG08571.1


```
 CLUSTAL W (1.82) Multiple Sequence Alignments - Introns Inserted


Sequence 1: NCU02148.1	627 aa
Sequence 2: MG02622.1	586 aa
Sequence 3: FG08571.1	623 aa
Sequence 4: AN4684.1	465 aa
Alignment Length: 661 aa
Number Identitical Residues: 248 aa
Alignment Score (without introns) 12181


MG02622.1 	-MPLPFLGRLNI2PEYVALLGSFFLVGLEAFIRVITLALP1-------------------
NCU02148.1	-MPVPFLGRLDV2TEYVALIGSFFLVGFEALIRVLTLALP~NTLITLFYRLSRRLFNRWT
FG08571.1 	-MAIPFIGRLRP2YEYFALVGSFILVGLEAVIRVLTLALP1-------------------
AN4684.1  	MARVPFIGRLFW~FEYLTLFGSLILVLLEWIIHIITFCLP1ELIINFCYERSKTIFNLVI
          	   :**:***    **.:*.**::** :* .*:::*:.** .   .     :    .   

MG02622.1 	-------------~VSTSIRDASDFVELCALNGYQAEEHVVQTKDGYLLGLHRLAWRKGE
NCU02148.1	TPAQKRAEERRQP1VSEAVRNASDFVDLCALFGYTAEEHVVQTKDGYLLGLHRLAYKKGE
FG08571.1 	-------------~ISTSVRDAADFVELCRIWGYEAEEHIAQTKDGYLLGLHRLQWRKGE
AN4684.1  	TPERPEKQGRRKR~RASAVAHASDFAEICSIYGYEAEEHIVQTGDGYLLGLHRLPHRKGE
          	:.     .   .   : :: .*:**.::* : ** ****:.** **********  :***

MG02622.1 	EDQRVNSGPNSVQKRVVYLHHGLLMNSEVWVCQTDTNRSLAFVLVDQGFDVW0RYIWQLG
NCU02148.1	EDTKVNRGPNSIKKRVVYLHHGLLMNSEVWVCLTDEQRCLPFVLVERGFDVW0-----FG
FG08571.1 	EGQKVNYGPTSLKKKVIYMHHGLLMNSEVWVSLTNEQRCLPFELVEKGYDVW0-----FG
AN4684.1  	ESQTVNQGEGSTKKKVVYLHHGLMMCSEVWVCLTDEERCLPFQLVERGYDVW~-----LG
          	*.  ** *  * :*:*:*:****:* *****. *: :*.*.* **::*:***      :*

MG02622.1 	NNRGNKYSKKSINHSPASTAFWNFSIDEFAFHDIPDSIAYILETTDEKSLSYIGFSQGTA
NCU02148.1	NNRGNKYSKKSVHCSPMSQRFWNFSIDEFAFHDIPDSISYILETTGQPSLSYIGFSQGTA
FG08571.1 	NNRGNKYSKKSIHHSPTSNAFWDFSIDEFAFHDIPDSISYILDTTQQESLSYIGFSQGTA
AN4684.1  	NNRGNKYSKKSTRHSPLSNEFWDFSIDQFAFHDIPDSINYILDLTGQPSLSYIGFSQGTA
          	*********** . ** *  **:****:********** ***: * : ************

MG02622.1 	QAFASLAVNPRLNQQVNVFIALAPAMSPAGLSNRVVDALIKSSPSVLFLLFGRRAILSSA
NCU02148.1	QAFASLSIHPKLNDQVNVFIALAPAMSPAGLSNGIVDALVKASPQVLFLLFGRRSILSSA
FG08571.1 	QAFASLAIHPKLNNQVNVFIALAPAMAPAGLSNGIVDALVTASPSVLFLLFGRRSILSSA
AN4684.1  	QAFATLSIHPQLNQKIDVFVALAPAMAPARISNPVVDSLMKASPNFLFLLFGRRSILSST
          	****:*:::*:**::::**:******:** :** :**:*:.:**..********:****:

MG02622.1 	TMWETILYPPIFIKVIDVAVSFLFGWKTRNISTSQKLAAYPHLYSFTSTKSVVHWFQIIR
NCU02148.1	TMWQSILYPPIFAKLIDMGLSFLFNWKTQNISTSQKLAAYPHLYSFTSTKSVVHWFQIIR
FG08571.1 	TMWETILYPPIFSKLIDMGLSFLFNWQTLNISASQKLAAYPHLYSFTSTKSVVHWFQIIR
AN4684.1  	TMWQTILYPPIFMRIIDTSLSFLFNWKCKNISHDQKLAGYLHLFSFTSTKSVVHWFQIIR
          	***::******* ::** .:****.*:  *** .****.* **:****************

MG02622.1 	TKSFQMYDDDVQQPLSLSTSSRFTKVAKYPTRNIKTPIVLVYGGSDSLVDIKVMLRQLPP
NCU02148.1	TKSFQMFDDDVQPPLMLSTSSKYTKVAKYPTRNIKTPIVLVYGGIDSLVDIKVMLRELPT
FG08571.1 	NKCFQMYDDDVHQPISVTSTRKYSKVAKYPTRNIKTPIVLVYGGSDSLVDIKVMLKELPP
AN4684.1  	NRNFQFYDDEIYAPFSIVASERFYKPVKYPTKNIKTPIVLLYGGSDSLVDIDVMQKELPR
          	.: **::**::  *: : :: :: * .****:********:*** ******.** ::** 

MG02622.1 	QTVATEIPRYEHIDFLWARDVDAQVFRHVFDALDSFTGAEHTKEEYNRYRTARSSSISAS
NCU02148.1	QTVATEIPHYEHLDFLWARDVDAQVFQHVFDALESFTGAEHTKEEYERYRSARHVSLSAS
FG08571.1 	QTVATEIPHYEHLDFLWARDVDTQVFQHVFDALDSFTDAEHTKEEYDRYYVSRQESLLGS
AN4684.1  	GTTAKIITKYEHLDFLWASDVSELVFGHVFEALDRYGPTKRLPDG------SVNGLINGA
          	 *.*. *.:***:***** **.  ** ***:**: :  :::  :       :    : .:

MG02622.1 	-VNFRRHAHHDSVDTQADVTDETTATTTEAESDSGDRTLVNGRGGS--------------
NCU02148.1	-NSLRRFKPNKNGDNMAVDSDVSTAVAGSVHD--GEEQFIDADERQ--------------
FG08571.1 	GYAYARSQAHLGSESESSTLTPSLEGTNSIQLAPAPQPHRARETASGIPSPKNTTRHRVN
AN4684.1  	------------------------------------------------------------
          	                                                            

MG02622.1 	---RSRLRHQATEGLRSPSRGSEVSVGPASGGLNTNGTVASDGES------SSGSPARRR
NCU02148.1	---ERLGQIEAPHQARESPLTTSVGSLSSTSGSKLGGNILRRPNS------RPTSPMYPG
FG08571.1 	YSIDKIGDPPVTPTFQTEPAEPPAGQEQSEEGVQTEPTEPSAGQEQLDEGVRTPIAARVK
AN4684.1  	------------------------------------------------------------
          	                                                            

MG02622.1 	AGIQR-HTSGVSVDS--LRQGRGIRVGASRAVG-----GVTAAIAGGNSPSSGTD-----
NCU02148.1	SLDTR-FENGSRAESPSDRSPDSEYVGALPSVTPPKRTGTGASQMSLNSLRAGKGISLGA
FG08571.1 	AGVTRSGSVGSNISLDGMREGRGITIGASRAIGGVVTKSGASGTNGEGSPGRDSGSENKK
AN4684.1  	------------------------------------------------------------
          	                                                            

MG02622.1 	-----
NCU02148.1	SESP-
FG08571.1 	NKKNK
AN4684.1  	-----
          	
```
